# The Role of Liver Transplantation in the Treatment of Liver Metastases from Neuroendocrine Tumors

**DOI:** 10.1007/s11864-023-01124-w

**Published:** 2023-10-26

**Authors:** Davide Citterio, Jorgelina Coppa, Carlo Sposito, Michele Droz Dit Busset, Matteo Virdis, Isabella Pezzoli, Vincenzo Mazzaferro

**Affiliations:** 1 General Surgery and Liver Transplantation Unit, Fondazione IRCCS Istituto Nazionale Tumori, Via Venezian 1, 20133 Milan, Italy; 2 Department of Oncology and Hemato-Oncology, University of Milan, Milan, Italy

**Keywords:** Neuroendocrine liver metastases, Liver transplantation, Transplant oncology

## Abstract

Transplant oncology is a new field of medicine referred to the use of solid organ transplantation, particularly the liver, to improve prognosis and quality of life in cancer patients. In unresectable, liver-only metastases from neuroendocrine tumors (NETs) of the digestive tract, liver transplantation represents a competitive chance of cure. Due to the limited resource of donated organs, accurate patients’ selection is crucial in order to maximize transplant benefit. Several tumor- and patient-related factors should be considered. Among them, primary tumors with a low grade of differentiation (G1-G2 or Ki67 < 10%), located in a region drained by the portal system and removed before transplantation with at least 3–6 months period of disease stability observed before transplant listing, can be considered for transplantation. In case of NET located in the pancreas, extended lymphadenectomy should complement curative pancreatic resection. A number of other features are described in this review of liver transplantation for NET metastases. Comprehensive approach including various forms of non-surgical treatment and detailed planning and timing of total hepatectomy are discussed. Open issues remain on possible expansion of current criteria while maintaining the same long-term benefit demonstrated with the Milan NET criteria with respect to other non-transplant options, with particular reference to liver resection, peptide receptor radionuclide therapy, and locoregional and systemic treatments.

## Introduction

Neuroendocrine tumors (NETs) are rare tumors originating from the widespread neuroendocrine cells, and the majority of them is well differentiated with an indolent behavior. Primary NETs are mainly located in the gastroenteropancreatic (60%) and pulmonary system (25%) [1] and are usually diagnosed at a metastatic stage, being the liver the most commonly affected organ (40–93% of cases), followed by the lung (8–10%) and bone (12–20%). Neuroendocrine liver metastases (NELM) are a major prognostic factor, associated with a significantly reduced survival [2].

Surgical resection is the optimal approach for NELM, but radical resection is achievable only in a small proportion of patients since the pattern of liver involvement is often characterized by multiple and bilobar nodules, unresectable with curative intent. In selected patients with liver-only unresectable metastases, liver transplantation (LT) has demonstrated excellent long-term outcomes.

LT for malignancy is an evolving field, with some consolidated indications and other more innovative ones that are still under investigation. Historically, hepatocellular carcinoma (HCC) as a primary liver tumor was the first to be managed by means of organ substitution [3], and during the last decades, substantial advancements have been made on patients’ selection, management on the waiting list, and prioritization, leading to improved patients’ outcomes [4, 5].

Currently, HCC represents the most common indication for LT globally, since in most transplant centers, the proportion of HCC patients in the waiting list ranges between 15 and 50% [6–9]. From HCC, the indication for LT extended in a step-way fashion to other primary liver cancers such as cholangiocarcinoma, hepatoblastoma, and hepatic epithelioid hemangioendothelioma with satisfactory survival rates [10–12].

Considering secondary liver tumors, LT has been accepted for long time only in the case of NETs, following the positive results of several studies. On the contrary, LT for unresectable liver metastases from colorectal cancer has been abandoned following the first experiences published in the literature due to the very poor long-term outcomes reported [13, 14]. Things changed radically in 2013, the year of publication of the SECA (secondary cancer) trial, a pilot study from Norway reporting a 60% 5-year survival in 21 patients with unresectable LM from colorectal cancer undergoing LT [15]. This study showed that, despite a high rate of pulmonary recurrence, patients could achieve unprecedented long-term survival with respect to conventional treatment with chemotherapy. This study was followed by others from the same group reporting even better outcomes with more restrictive selection criteria, and many other transplant centers started their own protocols on LT in colorectal LM. The widening of malignant indications for LT opened the era of the so-called transplant oncology, a new field born from the fusion of surgical oncology and transplantation surgery. The issue of colorectal cancer LM in this field is particularly cumbersome because of the high prevalence of this cancer which is one of the “big killers,” epidemiologically much more frequent than NET, that are considered rare tumors. More than 30% of patients with colorectal cancer present with LM during the course of the disease, and resectability is around 10–20%. Currently, the number of patients eligible to LT is low because selection criteria are quite restrictive. Nevertheless, this indication may compete in the near future with NET patients for the allocation of organs outside “conventional” indications. Therefore, in this evolving scenario of transplant oncology with new indications coming into the field, the selection of NET patients for LT is of utmost importance to maximize patients’ survival benefit and justify the enlistment of these patients as Model for End-Stage Liver Disease (MELD) exceptions, considering the shortage of donated organs.

This article provides a critical overview of the role of LT in the treatment of NELM, from the selection criteria to be adopted to the results with respect to alternative therapies.

## Selection criteria for liver transplantation

The treatment of choice for NELM is surgical resection, as recommended by major guidelines. Particularly in G1-G2 tumors and in absence of extrahepatic spread, radical surgery is associated with excellent long-term outcomes [16•]. Advanced procedures such as staged hepatectomy, portal vein embolization, and associated liver partitioning and portal vein ligation for staged hepatectomy (ALPPS) should be implemented to achieve R0 resection. Unfortunately, a large proportion of patients presents with unresectable liver disease amenable only to LT as curative option. In the initial experiences, LT was applied as a salvage procedure for symptomatic patients and/or vey advanced cases; similarly to what happened for other oncologic indications to LT, the long-term results were unfavorable [17–19]. Nowadays, even if a high level of evidence on the role of LT in the management of NET is still lacking, the experience gained in the main transplant centers with active LT programs for NET patients made it possible to establish some guiding principles for patients’ selection, aiming at cure.

Firstly, removal of the primary tumor together with its regional lymph nodes should be accomplished before considering LT. This approach has three aims: first, to gather definitive information on the pathological status of the primary tumor (Ki67%, grading, T stage, N stage); second, to remove all the non-hepatic locations of the disease; third, to reduce the risks of peri-transplant morbidity (simultaneous resection of the primary tumor at the time of LT is in fact associated with dismal short and long term outcomes) [19–21]. Regarding the location of the primary tumor, only those draining in the portal system should be considered, because in other cases the risk of dissemination to other organs, such as bones or lungs, is too high.

The presence of extrahepatic disease is an absolute contraindication and must be accurately ruled out by means of conventional radiology together with Ga68 PET, since the latter has the highest sensitivity in this setting [22].

Looking at the biology of the tumor, only well- and moderately differentiated G1-G2 NET can be considered for LT, with some series reporting a Ki67 less than 10% as inclusion criteria [18–20, 23], because the differentiation grade has a heavy impact on prognosis. Another mean to select tumors with favorable behavior is to apply an observation period of 3 to 6 months after primary tumor removal before enlisting, since time is the best surrogate of tumor biology. This strategy is considered appropriate to prevent the “fast-track” effect, i.e., to transplant patients at high risk of post LT recurrence [24, 25].

The tumor burden is another key factor: a liver involvement of more than 50% is associated with poor prognosis both after liver resection and after transplantation [26, 27]. In a series from Le Treut et al., hepatomegaly, defined as an explanted liver volume of more than 20% greater than the standard liver volume, was an independent predictor of poor prognosis [17].

Finally, age < 60 years at transplantation should be kept as a relative criteria because it has been demonstrated that transplant benefit increases over time and older age is correlated with adverse outcomes [21, 25].

The Milan criteria, proposed by our group taking into account the aforementioned factors, lead to an excellent 5- and 10-year post-LT survival (97.2% and 86.9%, respectively) in a prospective study [25].

These criteria have been endorsed in the USA by the Organ Procurement and Transplantation Network (OPTN), to approve a LT candidate for NELM as MELD exception [28] and in Europe by the European Society for Medical Oncology (ESMO) guidelines [29••]. According to the European Neuroendocrine Tumor Society (ENETS) guidelines, published in 2016, LT is not recommended as treatment option for advanced NET, but it may be considered in highly selected patients with carcinoid syndrome or other functional NET, and extended liver disease, refractory to multiple systemic treatments [30]. According to these guidelines, LT should still be considered as a salvage procedure in case of failure of conventional treatments. New indications may be coming from the ENETS guidance paper on NELM, currently under development. The North American Neuroendocrine Tumor Society (NANETS) guidelines propose both the Milan criteria and the abovementioned ENETS guidelines for patients’ selection and consider LT an option with good results for some patients, but “the scarcity of organs and the requirement that patients generally have favorable tumor biology (and thus may also be candidates for cytoreduction) have limited its use” [31••] (Table 1).

**Table 1:** Summary of the current recommendations for liver transplantation in neuroendocrine tumors across guidelines

ESMO [29••]	Liver transplantation may be a valid option in very selected patients with unresectable LMs when the following criteria are met: absence of extrahepatic disease, histological confirmation of a well-differentiated (G1/G2, Ki-67 < 10%) NET, previous removal of primary tumor, metastatic diffusion < 50% of the total liver volume, stable disease in response to therapy for at least 6 months before transplant consideration, and age < 60 years
UNOS/OPTN [28]	Resection of primary malignancy and extra-hepatic disease without any evidence of recurrence at least six months prior to MELD exception request Neuroendocrine liver metastasis limited to the liver, bilobar, not amenable to resection Consider for exception only those with a NET of gastroenteropancreatic origin tumors with portal system drainage. Note: Neuroendocrine tumors with the primary located in the lower rectum, esophagus, lung, adrenal gland, and thyroid are not candidates for automatic MELD exception Lower-intermediate grade following the WHO classification. Only well differentiated (low grade, G1) and moderately differentiated (intermediate grade G2). Mitotic rate < 20 per 10 HPF with less than 20% ki 67 positive markers Tumor metastatic replacement should not exceed 50% of the total liver volume Negative metastatic workup should include one of the following: a. Positron emission tomography b. Somatostatin receptor scintigraphy c. Gallium-68 (68 Ga) labeled somatostatin analogue 1,4,7,10-tetraazacyclododecane-N, N′, N″,N′″-tetraacetic acid (DOTA)-D-Phe1-Try3-octreotide, or other scintigraphy to rule out extra-hepatic disease, especially bone metastasis
ENETS [30]	Liver transplantation may be considered in highly selected patients with carcinoid syndrome or other functional NET, and extended liver disease, refractory to multiple systemic treatments
NANETS [31••]	Liver transplantation is controversial but may be an option for some patients if the Milan and ENETS criteria are met

*ESMO* European Society for Medical Oncology, *UNOS* United Network for Organ Sharing, *OPTN* Organ Procurement & Transplantation Network, *ENETS* European Neuroendocrine Tumor Society, *NANETS* North American Neuroendocrine Tumor Society, *MELD* Model for End-Stage Liver Disease

Globally, some issues remain open in the context of LT for NET, in particular the optimal timing for transplantation along the natural history of the disease, the actual benefit of LT in the era of molecular-targeted therapies and PRRT, and the indications for living donor LT.

## Liver transplantation vs. liver resection

**Fig. 1:**
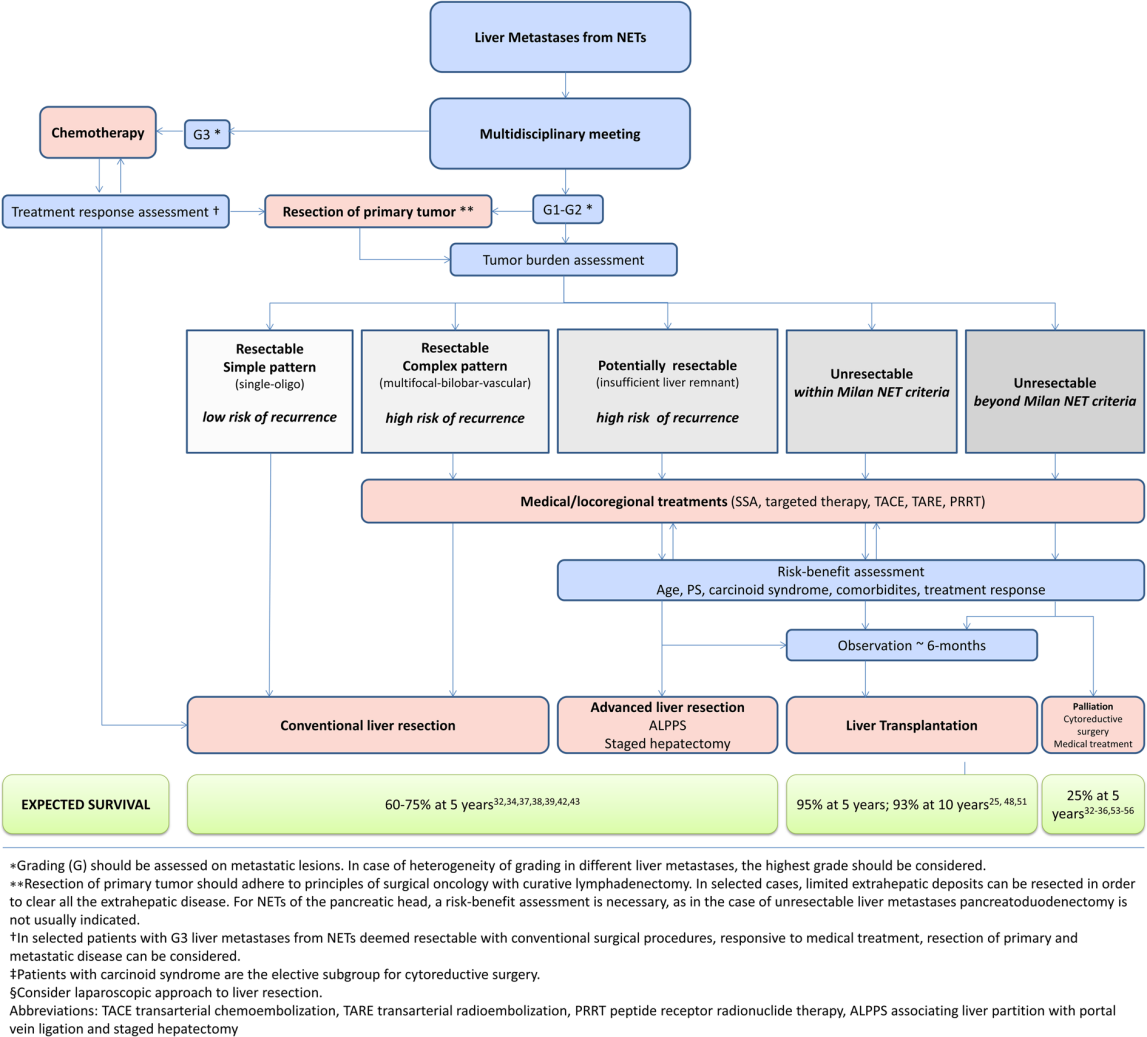
Treatment algorithm and expected survival for patients affected by liver-only metastases from gastroenteropancreatic neuroendocrine tumors (GEP-NETs).

As reported in Fig. 1, complete resection of metastatic disease offers a real chance of long-term survival in patients with NELM, even though cure is rare [32•]. Resection is associated with 5-year overall survival rates of 60–75%, as opposed to 25–65% of medical treatments [33–36]. In a meta-analysis collecting 1108 patients, those who underwent liver surgery for NELM had median 1-, 3-, and 5-year OS rates of 92.7%, 76.9%, and 67.5%, respectively, all superior to the equivalent OS rates of the non-resection groups (77.3%, 40.9%, and 26.6%, respectively; *p* < 0.001) [37]. Generally, radical resection is feasible in 45–55% of patients, and nearly all patients experience subsequent disease recurrence [38, 39••]. This disease behavior has been attributed to the high probability of preoperative understaging of the actual number of metastases, which may have miliary and ubiquitous distribution [40]. In an international multicentre study promoted by the Johns Hopkins University School of Medicine and published in 2010, the recurrence rate was 94% [34]. Similarly, the Mayo Clinic group reported an overall recurrence rate of 84% at 5 years and of 76% in the subpopulation of patients with R0 surgery [41]. A French group in 2010 reported the results of a study carried out analyzing thin-layer pathological slicing derived from 11 major hepatectomies for NELM, after an in-depth preoperative study with scintigraphy for somatostatin receptors, MRI with contrast medium, CT with contrast medium, and ultrasound. The analysis confirmed that the actual burden of disease can be 50% higher than the preoperative assessment [40]. The optimal width of the resection margin in case of resection is still a matter of debate [34, 42, 43], while it is widely recognized that R0 resections grant a better OS than R1, as reported by the Mayo Clinic group [41]. Even complex staged surgical procedures such as two-stage hepatectomies or ALPPS can be performed in optimal surgical candidates and with well-differentiated diseases, mainly in case of type II involvement according to Frilling et al. (i.e., isolated metastatic bulk with smaller deposits involving both hemilivers) [44, 45, 46•, 47]. Despite technical advancements and improved perioperative management, ALPPS remains a challenging surgical procedure, burdened by high morbidity also in NET patients (overall and grade ≥ 3b Dindo-Clavien 52% and 29%, respectively) [46•]. The incidence of recurrence being so high, repeated resections for relapse after radical surgery should be performed in patients with limited liver disease and are considered safe and feasible [30, 38, 39••]. Patients at greatest risk of recurrence are those with synchronous disease at onset, those with extensive liver involvement at the first radical treatment, those who have had R1 resections, and those who have undergone concomitant liver ablative treatments. Approximately 45% of patients with recurrent metastases are thought to be treatable with repeated resections, and it appears that this goal should be pursued, given that the 5-year survival of these patients remains significantly higher than those who are treated with medical or locoregional therapies [30, 38, 39••].

In the literature, there are no prospective comparative studies between LT and resection. A retrospective study published in 2021 from our group compared a series of 48 patients transplanted for NELM with a group of 56 patients submitted to radical resection, all within the Milan criteria for LT and treated with curative intent after removal of the primary tumor. Resection was offered to patients considered radically resectable, transplantation to unresectable ones. After a median follow-up of 13 and 10 years, respectively, the 3-year, 5-year, and 10-year survival rates were 98%, 95%, and 93% for LT and 92%, 90%, and 75% for LR (*p* = 0.007) and the 3-year, 5-year, and 10-year DFS rates were 84%, 75%, and 52% for LT and 49%, 33%, and 18% for LR (*p* < 0.001). The median disease-free interval between liver surgery and recurrence was longer for the LT group (78 vs. 24 months, *p* < 0.001). Transplant patients showed a tendency for multisite recurrence, while resected patients for intrahepatic recurrence. Survival after recurrence was similar in both groups. This study might suggest that any patient with disease presentation meeting the Milan criteria may achieve a significant survival advantage with LT [48••] Given the worldwide donor scarcity, the good performance status of the majority of candidates, the availability of alternative therapeutic options for these tumors, and their long natural history even if untreated, the option of LT should be carefully balanced and proposed under restrictive criteria, also considering alternative sources of donation such as live donor liver transplantation [49••, 50]. More recently, Eshmuminov et al. published a multicenter retrospective analysis of 455 patients; 230 submitted to liver resection and 225 to liver transplant with a median postoperative follow-up of 97 months. PFS and OS were evaluated, as well as risk factors in relation to outcome. After propensity score matching, the median OS was 205 months for transplant recipients and 120 months for patients undergoing R0 resections, with an OS of 75% and 68.3% at 5 years, respectively (*p* = 0.015). In a sub-analysis considering patients within the Milan-NET criteria and favorable tumor biology (i.e., G1-Ki67 < 5%), liver transplantation still offered better OS and RFS compared to radical resection. This benefit was lost for patients transplanted outside the Milan criteria. The data therefore demonstrate that the benefit of LT relies on adherence to selection criteria, most notably a low-grade tumor biology [51••].

Another retrospective study including 238 patients undergoing LR for NELM reported a 5-and 10-year OS of 83.3% and 71.4%, respectively, for the 28 patients of the series meeting Milan criteria. Patients with more favorable clinic-pathological characteristics (i.e., G1 patients, patients undergoing minor LR, patients with 1–2 NELM, and/or patients with tumor size < 3 cm) achieved a 5-year OS > 90%. These data suggest that resection with radical intent should be the first option for patients with resectable NELM, while LT should be reserved to patients with unresectable NELM, particularly in case of type III pattern of NELM according to Frilling (disseminated bilobar tumor growth) [47].

## Liver transplantation vs. locoregional and systemic therapies

In patients with NELM deemed unresectable due to extensive liver involvement, proximity to crucial vascular and biliary structures, or insufficient liver remnant, systemic therapy with possible addition of liver-directed treatments or targeted radionuclide therapy is the mainstay of management for most cases, while LT is still considered as investigational in this setting [30]. While the benefit of somatostatin analogues (SSAs) [35, 52], everolimus [53], sunitinib (for pancreatic NET) [54], peptide receptor radionuclide therapy (PRRT) [55], and chemotherapy (capecitabine + temozolomide) [56] on PFS of advanced NET patients have been confirmed in several randomized studies, available data on locoregional treatments and PRRT specifically in the setting of liver-only disease mainly lie in retrospective series or phase 2 studies. As for trans-arterial embolization (TAE) or chemoembolization (TACE), response rates (ORR) around 45–80% with median PFS of 15–30 months have been reported; studies on PRRT demonstrate lower ORR (15–40%) but satisfactory long-term disease control with PFS around 20–35 months [57]. Although retrospective comparisons of patients treated with medical therapies or LT highlight the advantages of the transplant option, comparative studies between LT and systemic or locoregional strategies aimed at evaluating the survival benefit or improvement in quality of life are scarce [58]. Furthermore, the benefit of LT should be evaluated according to recent improvements in non-surgical multimodal treatment of NELM, because most of the transplant series belong to a time when PRRT and targeted agents were not available.

As the long course and rarity of disease impair the feasibility of randomized studies, propensity matched and survival-benefit analyses may provide the best evidence. Nevertheless, the only comparative report published at present is a matched cohort study by Mazzaferro et al., enrolling two groups of patients with NELM undergoing either LT or optimal medical treatment with a combination of SSAs, targeted agents, chemotherapy, liver-directed therapies, and PRRT. Despite similar primary tumor features and liver disease burden, the survival benefit of the transplant group increased over time and exceeded 3 years after 10 years from LT [25].

Although the excellent survival outcome of LT has been widely confirmed in other single-center and multicenter series and registry reports, high rates of disease recurrence around 30–50% have been shown in most series [19, 20, 25, 59–67]. Such figures are partially due to heterogeneity of criteria used for LT but question the role of LT as the only potentially curative treatment for patients with otherwise unresectable NELM. However, recent data highlight that, even in patients undergoing recurrence, favorable long-term outcome is possible, particularly in those recurring more than 2 years after LT and in whom an aggressive surgical treatment is undertaken [68]. Whether this is the result of optimal patients’ selection, with inclusion of patients with favorable biology who would obtain similar outcomes with non-surgical treatments, or whether the achievement of radical resection of disease provides superior outcomes is still a matter of debate.

Another aspect to be considered is the different quality of life between patients treated by LT, those undergoing lifelong immunosuppressive therapy, and those managed by non-surgical strategies, with continuous need of medical, locoregional, and targeted treatments. Despite the high recurrence rates following LT, with very heterogeneous RFS reflecting high heterogeneity of selection criteria, the reported time to recurrence often exceeds 5 years [17, 19, 21, 25, 60, 62]. The achievement of cure, although temporarily, in a relevant fraction of patients, may improve quality of life for a long time. Even after recurrence, radical treatments are possible in a considerable subset of patients, and for those not amenable to repeat surgery, the time gained by LT without need for any oncological treatment may delay the onset of treatment-refractory disease once non-surgical strategies are undertaken, thus allowing for prolonged survival [68]. Although more definitive answers will need more robust comparative data in order to highlight the potential advantages of LT in terms of both survival and quality of life, indirect available evidence seems to support that a combination of patients’ selection and aggressive surgical treatment, encompassing LT for unresectable patients, may enhance the chances of long-term survival.

A final issue that still needs to be clarified concerns the indication to LT in patients with stable NELM while on medical treatment. Based on previous reports suggesting a better outcome of LT in such cases, a delay of at least 6 months was proposed, in order to assess tumor progression before listing [25]. However, it has recently been highlighted that asymptomatic patients with favorable tumor biology and stable disease may be safely managed with systemic therapy, possibly integrating locoregional options in case of slow-progressing lesions [69]. Given the extremely good outcomes obtained with optimal medical treatments, the transplant benefit may be marginal, and such patients may never require a LT during their disease course. On the other hand, patients with NELM refractory to medical and locoregional treatments due to more aggressive features, despite lower expected post-LT outcomes, may actually derive a greater survival benefit compared to non-transplant options. At present, evidence in this regard is insufficient to drive any meaningful conclusions on the optimal timing for LT in NELM and on the transplant benefit according to disease presentation and course.

## Open issues and future perspectives

Liver transplantation is an excellent opportunity of cure for patients with unresectable NELM. The criteria for selecting patients with NELM to LT take into account several tumors’ and patients’ related factors: these criteria have been established and applied since 2007 in order to identify biologically favorable tumors and thus to restrict the indication to those patients who are less likely to recur after LT and to best benefit in terms of long-term survival. Since then, several things have changed both in the transplant oncology scenario and in the knowledge and treatment of NETs, with potential influence on selection criteria to LT in the next future. Post-transplant tumor recurrence is fatal in most cases when the indication to LT is primary liver tumor [70], and therefore recurrence-free survival has been for long a reliable endpoint in transplant oncology. In the last 10 years, several studies have demonstrated excellent survival outcomes in patients undergoing LT for liver metastases from colorectal cancer (CRLM), despite very high post LT recurrence rates [71]. These results have progressively led the transplant community to reconsider the role of LT not exclusively as a chance for cure, but also as the possibility for excellent long-term palliation in metastatic, chemoresponsive tumors. Moreover, several treatments of metastatic NETs have become available and have shown effectiveness also in case of post-transplant recurrence. Consequently, today it is conceivable to consider an expansion of selection criteria accepting the risk of higher recurrence rates. This expansion, in order not to be detrimental in terms of overall survival, is likely to be hypothesized toward those features that are not directly linked to tumor aggressiveness: higher tumor burdens (i.e., > 50% liver involvement, or symptomatic patients), extraintestinal primary tumors (i.e., bronchial carcinoids), higher patients’ age. Conversely, patients in progression under treatment or high-grade tumors are still to be maintained out of transplant consideration, since these features of biological aggressiveness might have a direct influence on recurrence and post-recurrence survival.

On the opposite, also some expansion of LT in the field of resectable NELM can be foreseen. Resectability of liver metastases depend on factors related to patient conditions, to anatomical limitations (i.e., technical resectability) and to tumor characteristics (i.e., oncologic resectability, impacting on long-term outcomes after a R0 liver resection) [72, 73]. Even if technically resectable, NELM patients with very high tumor burden experience liver recurrence in almost 100% of cases [38]; moreover, patients with extensive liver disease often require highly demolitive liver resections that might result in severe postoperative morbidity [46•]. Therefore, in the context of resectable NELM with high tumor burden — requiring major hepatectomies or at high risk of R2 liver resection — LT can be proposed as primary curative treatment with higher chances of survival benefit than liver resection. Conversely, for patients with more limited disease, liver resection should be the primary treatment choice, and LT can be considered at recurrence as a “salvage” treatment. In this regard, however, most LT series on NELM include “naïve patients” in terms of previous liver resections, and literature on the “salvage LT” strategy is still scarce.

In conclusion, LT offers excellent long-term outcomes in selected patients with NELM. Neuroendocrine tumors are slow progressing but lethal cancers: the benefit of LT with respect to systemic or locoregional therapies is appreciated on the long term, and it is unlikely that randomized studies will be designed to provide definitive evidence. Modern liver transplant is a procedure with acceptable perioperative morbidity and low mortality that can offer a health-related quality of life (HRQoL) fairly comparable with age-matched groups [74]. This further aspect should be kept for potential referrals to LT: patients with remaining liver metastases often suffer from hormonal symptoms or side effects of multimodal treatments that are scarcely tolerated [75], for which liver transplant might be a long-term relief.
